# Evaluating the effects of storage conditions on dry matter loss and nutritional quality of grain legume fodders in West Africa

**DOI:** 10.1016/j.anifeedsci.2020.114419

**Published:** 2020-04

**Authors:** D.B. Akakpo, I.J.M de Boer, S. Adjei-Nsiah, A.J. Duncan, K.E. Giller, S.J. Oosting

**Affiliations:** a Animal Production Systems Group, Wageningen University & Research, P.O. Box 338,6700 AH Wageningen, the Netherlands; b Plant Production Systems Group, Wageningen University & Research, P.O. Box 430, 6700 AK Wageningen, the Netherlands; c International Institute of Tropical Agriculture, P.O. Box TL 06, Tamale, Ghana; d International Livestock Research Institute, P.O. Box 5689, Addis Ababa, Ethiopia; e Forest and Horticultural Crops Research Centre, Kade, University of Ghana, Legon, Ghana; f Global Academy of Agriculture and Food Security, The Royal (Dick) School of Veterinary Studies and The Roslin Institute, University of Edinburgh, Easter Bush Campus, Midlothian, EH25 9RG, UK

**Keywords:** Crop residues, Storage, Fibre, Crude protein, Aflatoxin, *in-vitro* digestibility

## Abstract

Feed scarcity is a major challenge for livestock production in West Africa, especially during the dry season when grass quality and quantity on grazing lands are inadequate. In the dry season, crop residues are a key source of livestock feed. The residues of grain legumes, also known as grain legume fodders (GLFs), are stored and traded for feeding in the dry season. The objectives of our experiment were to evaluate the effects of storage conditions and duration on dry matter (DM) and nutritional quality of GLFs, and to assess the risk of aflatoxin in stored groundnut fodder. The experiment was designed as a factorial trial with 18 treatment combinations with four replicates (4 farms). The treatments included: 3 types of GLFs (cowpea, groundnut and soybean fodder), 3 types of storage locations (rooftop, room and tree-fork) and 2 types of packaging (packed in polythene sacks and unpacked but tied with rope). Over a 120 day storage period, DM quantity reduced by an average of 24 % across all storage conditions, showing a range from 14 % in the best condition (sacks and rooms) to 35 % in the worst condition (bundles tied with rope and stored on rooftops or tree-forks). Soybean fodder had no leaves, the lowest crude protein content (CP) and organic matter digestibility (OMD), and the highest content of cell wall components compared to cowpea and groundnut fodder. These nutritional quality parameters in soybean fodder hardly changed during storage. Cowpea and groundnut fodder showed a decrease in leaf-to-stem ratio (LSR), CP and OMD, and an increase in the content of cell wall components during storage, but their nutritional value remained better than that of soybean fodder. Storage in sacks resulted in less DM loss, in less reduction of LSR and in a smaller increase of the content of cell wall components than storage of bundles tied with rope. Our study shows that the DM loss, the decrease in LSR, and the increase in the content of cell wall components can be prevented partly by storing GLFs in sacks instead of tying bundles with rope, and to a minor extent by storing in rooms instead of in the open air. Aflatoxin was not detectable in the groundnut fodder samples. Our results highlight that attention to storage conditions can improve the feeding value of GLFs which are key for livestock nutrition during the dry season.

## Highlights

• Nutritional quality and dry matter quantity of grain legume fodders (GLFs) declined with increasing duration of storage.
• GLFs stored in sacks had better nutritional quality and less dry matter loss than stored GLFs tied with rope.
• Decline in nutritional quality is drastic in leaf fractions than in stem fractions of GLFs during storage.
• Aflatoxin was not detected in groundnut fodder during storage in the dry season.

## 1 Introduction

Feed scarcity and high feed cost are major challenges for livestock production in West Africa, especially during the dry season (Ayantunde et al., 2014; FAO, 2014). Natural pasture and crop residues represent the majority of the feed for ruminants in West Africa. The importance of crop residues in smallholder systems in West Africa is increasing for two main reasons. First, natural pastures on communal lands are reducing due to the conversion of rangelands to croplands to feed the increasing human population. Second, crop residues can be traded and can contribute to mitigate feed shortages or create additional income in a prolonged dry season. The residues of grain legumes, also known as grain legume fodders (GLFs), such as groundnut and cowpea haulms, are intensively traded (Ayantunde et al., 2014; Konlan et al., 2018; Samireddypalle et al., 2017). In northern Ghana and other sub-Saharan countries, such as Nigeria, Burkina Faso, Mali and Niger, GLFs are harvested, dried and stored, and used by farmers or sold to other livestock farmers, fatteners and traders. Sale of GLFs is a source of additional income to farming households. GLFs have better nutritional quality than cereal residues, such as maize and rice straw (López et al., 2005; Schiere et al., 2004). GLFs show good results when used as supplementary or sole feed for the fattening of ruminants in the region (Ayantunde et al., 2007; Dada et al., 1999; Larbi et al., 1999).

In northern Ghana, feed availability to animals increases after crop harvest, whereas a shortage occurs in the dry season, and this shortage becomes critical towards the end of the dry season, i.e. from February to April (Konlan et al., 2018). To ensure feed supply and to secure prices for GLFs in this critical period of the dry season, farmers and middlemen store GLFs till the late dry season from January to April. During the storage of GLFs, the nutritional quality is not checked before use or before marketing to other buyers. Even though storage aims to preserve the quality and quantity of fodders for later use, losses of nutrients during the storage process have been reported, particularly in crude protein content (Lemus, 2009; Guerrero et al., 2010). According to Guerrero et al. (2010) and Feyissa et al. (2014), factors, such as sunlight, heat, and precipitation, affect the quality of forages during storage. Another quality factor of concern is the development of mould during storage, which may lead to mycotoxin contamination. Considerable variability in mycotoxin occurrences and concentration levels has been reported in forages, which were attributed to environmental and forage management related factors (Gallo et al., 2015). These factors can be controlled by managing storage conditions. Little is known, however, about the impact of different storage conditions on the dynamics of nutritional quality and development of aflatoxin in GLFs during storage. Therefore, the objectives of the present study were to evaluate the effects of storage conditions and duration on dry matter and nutritional quality of GLFs and to assess the risk of aflatoxin in stored groundnut fodder.

## 2 Materials and methods

### 2.1 Source of grain legume fodders and experimental design

The study was conducted in four villages (i.e. Tansia, Tetauko, Kaadi, and Kupalgoga) in Binduri district (10°56′01.6″N, 0°18′53.7″W) in the Upper East Region of Ghana during the dry season (December 2015 to April 2016). This district is located in the northern Guinea Savanna (NGS) ecological zone, which is dominated by monocrops of maize, sorghum and millet that benefit greatly from rotation with grain legumes (Woomer et al., 2013). In this district, like other districts in NGS, farmers experience feed shortages during the long dry season, and GLFs can contribute to mitigate these feed shortages (Amole et al., 2014). The present study used harvested fodder from an earlier study about the effect of rhizobium inoculation and phosphorus fertilization on grain and fodder yield and quality of three grain legume crops: cowpea (*Vigna unguiculata* (L) Walp), groundnut (*Arachis hypogaea* L.) and soybean (*Glycine max* (L) Merr). Details of this agronomic trial were described by Akakpo et al. (2020). One farmer was selected in each village to host one replicate of the present study on his or her farm. Farmers could only participate if they had facilities to store GLFs, i.e. a rooftop, a storeroom (indoors) and mature live trees with forks suitable for holding a substantial volume of GLF. Only trees, such as neem (*Azadirachta indica* A. Juss.) and shea (*Vitellaria paradoxa* C. F. Gaertn), that were located within 20 m radius of the homesteads, were selected.

**Fig. 1:**
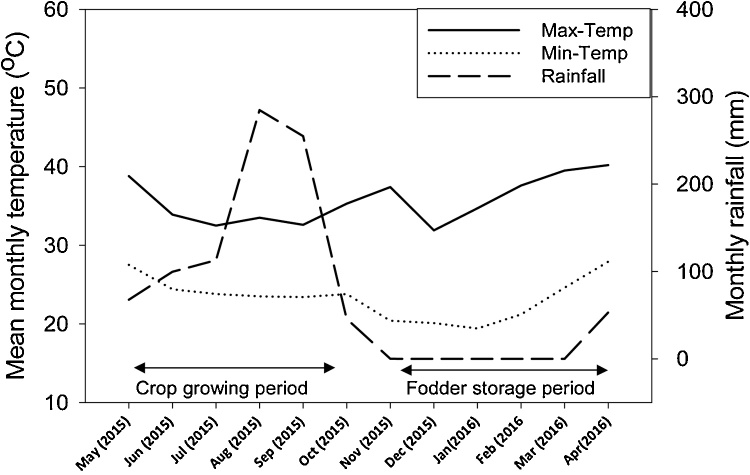
Mean monthly maximum and minimum temperature and monthly rainfall in the Binduri district during the experimental period (2015–2016). The arrows show the duration of the crop growing period and fodder storage period.

The weather data recorded at the Manga station of the Savanna Agricultural Research Institute (SARI) in the district indicated that the average annual minimum and maximum temperatures of the area were 23.3 and 36.7 °C, respectively with a mean of 30.0 °C. During the study year, the total annual rainfall was 919 mm, but there was no rainfall during the study (storage) period from December 2015 to April 2016 (Fig. 1).

The experiment was designed as a 3 × 3×2 factorial trial with 18 treatment combinations replicated four times in different villages (farms). The treatments included: 3 types of GLFs (cowpea, groundnut and soybean), 3 types of storage locations (rooftop, room and tree-fork), and 2 types of packaging (3 kg of GLFs bundled and packed in polythene sacks or unpacked but tied with rope). For each treatment combination, five bundles were used as an experimental unit.

At the time of harvest at each farm, fodders of each crop were collected on one heap and thoroughly mixed and left to dry for six days to attain constant weight. Per fodder heap, about 20 handfuls of samples were taken, pooled and mixed. Of this pooled sample three sub-samples of 200 g were taken for initial quality evaluation (Table 1) at the start of storage, which is also referred to as pre-storage quality. The sampled fodders were separated into leaf and stem fractions for groundnut and cowpea but not for soybean fodder, which consisted only of stems and threshed pods at harvest. After the six days of drying, each heap was mixed again and bundled in 3-kg weights. The bundles were either packed in 104 cm × 60 cm size polythene sacks or unpacked but tied with rope. The packaged and tied fodders were assigned to the storage locations according to the experimental design.

**Table 1:** Pre-storage leaf-to-stem ratios and nutritional composition and organic matter digestibility of leaf and stem fractions of cowpea, groundnut and soybean fodder.

Nutritional Parameter	Botanical	Cowpea	Groundnut	Soybean^1^
	fractions			
Leaf-to-stem ratio (LSR)		0.42	0.49	0
Crude protein (CP; g kg^−1^)	Leaf	165	180	
	Stem	162	165	97
Organic matter digestibility^2^ (OMD; g kg^−1^)	Leaf	737	677	
	Stem	746	644	548
Ash (g kg^−1^)	Leaf	144	151	
	Stem	150	150	80
Neutral detergent fibre (NDF; g kg^−1^)	Leaf	465	397	
	Stem	432	446	652
Acid detergent fibre (ADF; g kg^−1^)	Leaf	319	368	
	Stem	323	397	550
Acid detergent lignin (ADL; g kg^−1^)	Leaf	85	90	
	Stem	78	97	105
Cellulose (g kg^−1^)	Leaf	234	278	
	Stem	245	300	445
Hemi-cellulose (g kg^−1^)	Leaf	146	29	
	Stem	109	49	102

1 Soybean fodder contained no leaf in this study. ^2^*in-vitro* organic matter digestibility.

The stored fodders were weighed, and samples were taken monthly (30 days interval) for laboratory analyses. At each sampling time, about 40 g of fodder from each of the five bundles in each treatment were carefully sampled. For estimation of dry matter loss, we corrected for the quantities removed during sampling. Each sample was carefully separated into leaf and stem fractions. The fractions were weighed, placed in paper bags, labelled and oven-dried at 70 °C for 48 h to determine dry matter. The dried samples were ground to pass through a 1 mm screen with a laboratory hammer mill at the Soil Chemistry Laboratory of the Savana Agricultural Research Institute (SARI) – Nyankpala, Ghana. The ground fodder samples were stored at ambient temperature and later air-freighted to the animal nutrition laboratory of International Livestock Research Institute – Ethiopia for analyses. The samples were freighted under the permission (Permit No.12113) of the Ministry of Agriculture and Natural Resources in Ethiopia.

### 2.2 Fodder quality and aflatoxin analysis

Fodder samples were analysed for chemical composition and nutritional traits using conventional chemistry and Near Infrared Reflectance Spectroscopy (NIRS). The conventional chemical analysis implied quantifying the ash/organic matter (OM), dry matter (DM) and crude protein (CP) content and neutral detergent fibre (NDF) content, according to the methods described in AOAC (1990). The *in-vitro* organic matter digestibility (OMD) was assessed according to the in-vitro gas production procedure as described in Van Soest and Robertson (1985). Reference samples were selected and analysed by conventional wet chemical analysis. Results from the conventional wet chemical analysis were used to calibrate and update the NIRS equations to predict the nutritional composition for a wide range of legume forages, such as groundnut, cowpea and soybean. NIRS predictions were made using FOSS Forage Analyzer 5000 with software package WinISI, according to de Boever et al. (1995), and included predictions of ash, nitrogen (N) (crude protein = N × 6.25), neutral detergent fibre (NDF), acid detergent fibre (ADF), and acid detergent lignin (ADL) contents, and *in-vitro* organic matter digestibility (OMD). Hemicellulose was calculated as NDF – ADF and cellulose as ADF – ADL, according to Rinne et al. (1997). Finally, we calculated NDF residual as a percentage of pre-storage NDF in DM residue at each sampling time. Neutral detergent soluble (NDS) residual was calculated as 100 – NDF according to Mertens (2009).

Groundnut fodder samples were analysed for aflatoxin B1 and B2, produced by *Aspergillus flavus* and *A. parasiticus* and aflatoxin G1 and G2 which are produced by *A. parasiticus* and other related species. Aflatoxin analysis was conducted at the pathology and mycotoxin laboratory of the International Institute of Tropical Agriculture (IITA) - Nigeria according to the protocol of Cole and Dorner (1994). For aflatoxin analysis known positive reference samples were included in the protocol to ensure the method was working.

### 2.3 Calculations and statistical analyses

The experiment was designed to investigate the effect of storage location, duration and packaging on DM loss and nutritional quality of GLFs. First, we analysed the leaf and stem fractions of cowpea and groundnut to determine the nutritional quality differences between leaf and stem fractions (Table 1). Second, to analyse the data on whole-crop basis across all crops (cowpea, groundnut and soybean), we reconstituted leaf and stem fractions of cowpea and groundnut mathematically to represent the fodder (leaf and stem) by taking the weighted average of the fractions. The weighted averages were analysed together with soybean fodder (stems and pods) data which contains no leaves by using a mixed-effect analysis of variance model (Searle et al., 1992) in GenStat version 19 (VSN, 2017). In this model (Eq. 1 below), replications (block), crop, storage location, packaging types and duration were fixed factors, while blocks nested with crops within village were random factors.

(1) *Y_ijklmn_ = μ + B_i_ + C_j_ + L_k_ + P_l_ + (CLP)_jkl_ + BC_ijkl_+ D_m_+ (CLPD)_jklm_ +****ε****_ijklmn_*

where, *Y* = the response variable (DM residue, nutritional composition and OMD of the reconstituted fodder), μ = the overall mean, *B_i_* = effect of *i^th^* block (villages), C*_j_* = effect of *j*^th^ crop (*j* = cowpea, groundnut and soybean), *L_k_* = effect of *k^th^* storage location (*k* = rooftop, room, tree-fork), *P*_l_ = effect of *l^th^* packaging type (*l* = sack, tied), *(CLP)_jkl_* =interaction effect of the main factors: crop, storage location and packaging type, *D_m_* = effect of *m^th^* storage duration (*m* = day 0, 30, 60, 90, 120), *(CLPD)_jklm_* = all the interaction effects of the main factors with duration, *BC_ijkl_* = the random effect for crops within villages and ***ε****_ijklmn_* = residual error. *BC_ijkl_* and ***ε****_ijklmn_* were assumed to be normally distributed around zero with variance σ^2^_crop_ and σ^2^ε, respectively. The differences between means were tested using the Fisher’s least significance difference (LSD) test (P < 0.05).

The means of the data were subjected to polynomial regression analysis (Eq. 2) to determine the trend of changes in measured parameters due to the duration of storage according to the model:

(2) *Y = β_0_ + β_1_X + β_2_X^2^+ε.*

where *Y* = the response variable, *β_0_* = the intercept, *β_1_* = regression coefficient for linear effect of *X* on *Y*, *X* = duration (days), *β_2_* = regression coefficient for quadratic effect on *Y*, and *ε* = random error term. A linear model was fitted first to the fodder data, and if the linear term was significant, then a quadratic term was added.

## 3 Results

### 3.1 Effects of storage conditions on composition and nutritional quality

**Fig. 2:**
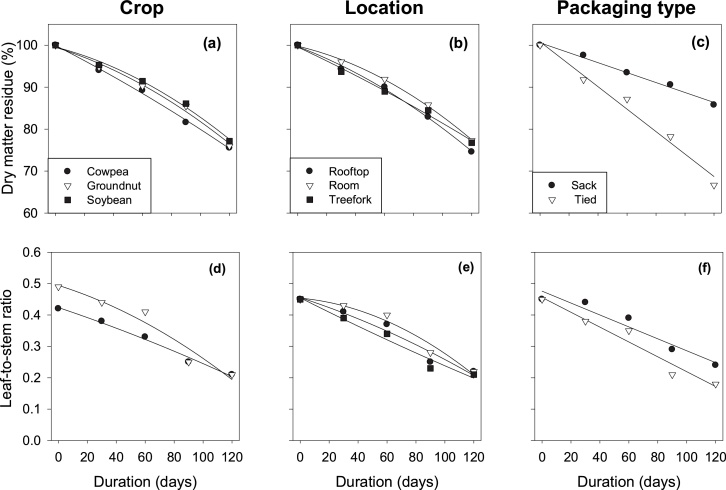
Effect of storage duration on dry matter loss (a, b, and c) and leaf-to-stem ratio (d, e, and f) among grain legume fodders at different storage locations and in different types of packaging. Soybean fodder contained no leaves.

The dry matter residues (DMR) of all crops reduced during storage for 120 days (Table 2; Fig. 2a–c). Soybean tended (*P* < 0.07) to have a higher mean DMR than cowpea and groundnut, whereas room storage tended (P < 0.07) to have a higher mean DMR than rooftop. Also, the mean DMR differed between fodder stored in sacks and tied fodder (Table 2). The rate of reduction of DMR differed among packaging types and equalled 0.12 % per day for fodder stored in sacks and 0.21 % per day for tied fodder (Table S1, Fig. 2c). On average, DMR decreased by 24 % across all storage conditions, with a range of 14 % for bundles packed in sacks and stored in rooms to 35 % for bundles tied with rope and stored on roofs or tree-forks (Fig. 2a, b and c).

**Table 2:** Leaf-to-stem ratio (LSR), dry matter residue (DMR) percentage, nutritional composition and organic matter digestibility of grain legume fodders stored at different storage locations and in different types of packaging for 120 days.

Treatments				Nutritional composition and organic matter digestibility of grain legume fodders (g kg^−1^ DM)						
	DMR (%)	LSR	CP	OMD	Ash	NDF	ADF	ADL	Cellulose	Hemi-cellulose
**Crop (C)**										
Cowpea	88.1^b^	0.32^b^	126^b^	699^a^	109^b^	521^b^	419^b^	86^c^	334^b^	101^a^
Groundnut	89.3^ab^	0.36^a^	148^a^	662^b^	132^a^	472^c^	433^b^	101^b^	332^b^	39^b^
Soybean	90.0^a^		99c	571^c^	95^c^	641^a^	543^a^	111^a^	432^a^	98^a^
*P*-value	0.07	<0.001	<0.001	<0.001	<0.001	<0.001	<0.001	<0.001	<0.001	<0.001
LSD	1.67	0.022	6.6	14.8	3.9	17.0	19.0	4.1	15.3	3.7

**Location (L)**										
Rooftop	88.4^b^	0.34^ab^	125	647	113	550	467	102^a^	366	82^a^
Room	90.2^a^	0.35^a^	126	645	112	536	460	97^b^	363	76^b^
Tree-fork	88.8^ab^	0.32^b^	122	640	111	549	469	100^ab^	369	80^a^
*P*-value	0.07	0.07	ns	ns	ns	ns	ns	0.07	ns	0.004
LSD	1.67	0.026	6.6	14.8	3.9	17.0	19.0	4.1	15.3	3.7

**Packaging (P)**										
Sack	93.5^a^	0.36^a^	126	649	113^a^	533^b^	455^b^	97^b^	358^b^	78
Tied	84.8^b^	0.31^b^	122	639	112^b^	556^a^	476^a^	102^a^	374^a^	81
*P*-value	<0.001	<0.001	ns	ns	0.05	0.002	0.009	0.001	0.017	ns
LSD	1.36	0.022	5.4	12.1	3.2	13.9	15.5	3.4	12.5	3.0

*P*-values for duration (D										
D	<0.001	<0.001	<0.001	0.02	<0.001	<0.001	<0.001	<0.001	<0.001	<0.001
D x C	ns	0.062	<0.001	<0.001	<0.001	ns	<0.001	<0.001	<0.001	<0.001
D x L	ns	ns	ns	ns	ns	<0.001	ns	0.031	ns	0.007
D x P	<0.001	ns	ns	0.059	ns	0.017	0.018	0.001	0.033	ns

CP = crude protein; NDF = neutral detergent fibre; ADF = acid detergent fibre; ADL = acid detergent lignin; IVOMD=*in-vitro* organic matter digestibility; ns = not significant. Means with different letters in a column of each treatment factor are significantly different (p < 0.05). LSR only applies to cowpea and groundnut fodders because soybean fodder contained no leaves.

LSR of groundnut and cowpea reduced during storage for 120 days, from 0.45 at pre-storage to 0.21 at the end of the storage (Fig. 2d). Mean LSR was higher in groundnut than in cowpea, but the difference in LSR between cowpea and groundnut reduced with storage duration and LSR of both fodders became similar at the end of storage. Room storage tended (P < 0.07) to have a higher mean LSR than storage on tree-fork (Fig. 2e). Also, the mean LSR differed between fodder stored in sacks and tied fodder (Table 2f). The rate of reduction of LSR differed among packaging types and equalled 0.0019 per day for fodder stored in sacks and 0.0024 per day for tied fodder (Table S1, Fig. 2f).

**Fig. 3:**
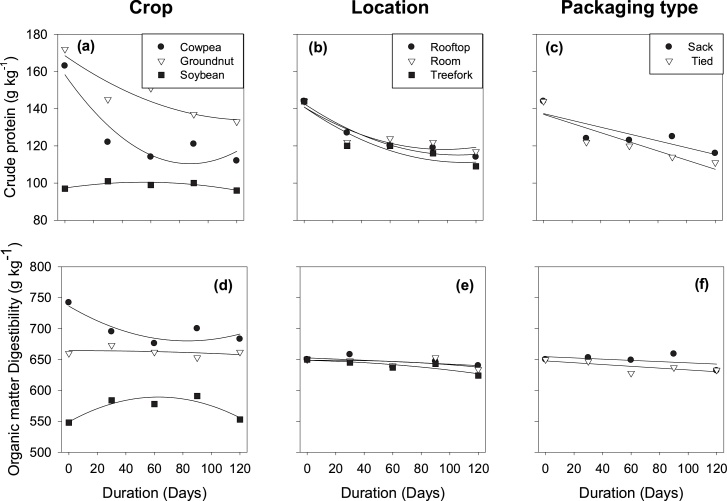
Effect of storage duration on crude protein (a, b, c) and in-vitro organic matter digestibility (d, e, f) of grain legume fodders at different storage locations and in different types of packaging.

Mean CP content differed among crops (Table 2). The mean CP content of groundnut was higher than that of cowpea, while soybean had the lowest CP content (Table 2). There was no effect of storage location and packaging type on CP content during storage. The CP content of GLFs declined rapidly in the first 30 days of storage and stabilized thereafter, with an interaction between duration and crop (Table 2; Fig. 3a). After 120 days of storage, CP content had reduced by 31 % in cowpea and by 21 % in groundnut (Table S1). During storage, the CP content of stem fractions of cowpea and groundnut reduced quadratically, but that of the leaf fraction remained relatively constant (Data not shown).

Mean OMD differed among crops. Cowpea had the highest OMD, followed by groundnut, while soybean had the lowest OMD (Table 2). There was a duration effect on OMD of GLFs with a significant interaction between duration and crop (Table 2). OMD of cowpea reduced quadratically, illustrating a decline in the first 30 days of storage and remaining relatively constant after that, whereas OMD of groundnut and soybean remained relatively constant during storage (Fig. 3d). During storage, the OMD of the stem fraction of cowpea reduced quadratically, but that of the leaf fraction remained relatively constant (Data not shown).

**Fig. 4:**
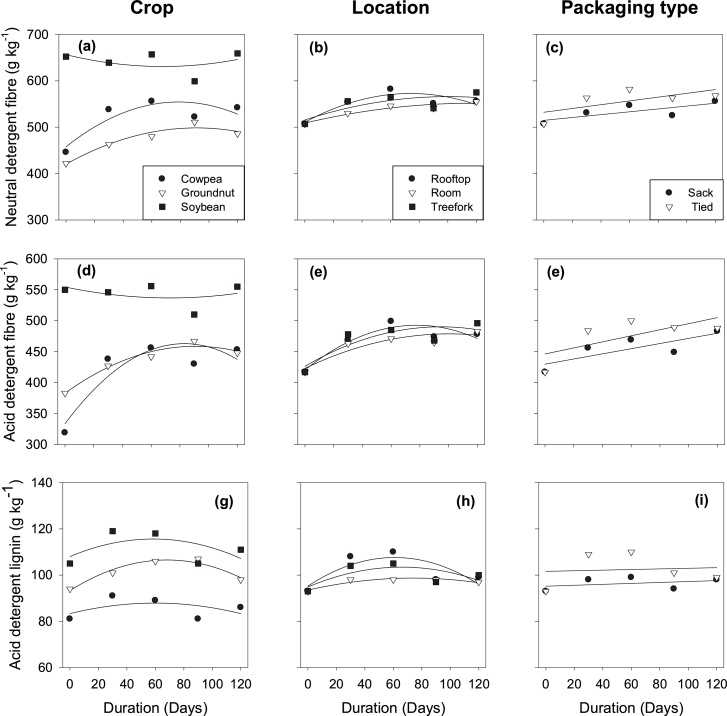
Effect of storage duration on the fibre content: neutral detergent fibre (a, b, c), acid detergent fibre (d, e, f) and acid detergent lignin (g, h, i) of grain legume fodders at different storage locations and in different types of packaging.

The mean ash content and cell wall components (NDF, ADF, ADL, cellulose and hemi-cellulose) differed among crops (Table 2). Soybean had the lowest ash content and, in most cases, the highest content of cell wall components compared to cowpea and groundnut (Table 2). Room storage had lower mean ADL and hemi-cellulose contents than storage on tree-fork and rooftop, whereas fodder stored in sacks had lower means for NDF and ADF and a higher mean for ADL than tied fodder. There was a duration effect on the ash content and cell wall components (Table 2 and Fig. 4) with some of these components showing significant interactions between duration and crop, duration and location and duration and packaging type. Noteworthy findings regarding these interactions are that NDF and ADF increased quadratically during storage for cowpea and groundnut fodder, but there was no change for soybean fodder. After 120 days of storage, NDF had increased by 22 % in cowpea and 15 % in groundnut (Table S2). Moreover, the rates of change in NDF and ADF were different between packaging type and showed a linear rate of increase (Table S2, Figs. 4).

**Fig. 5:**
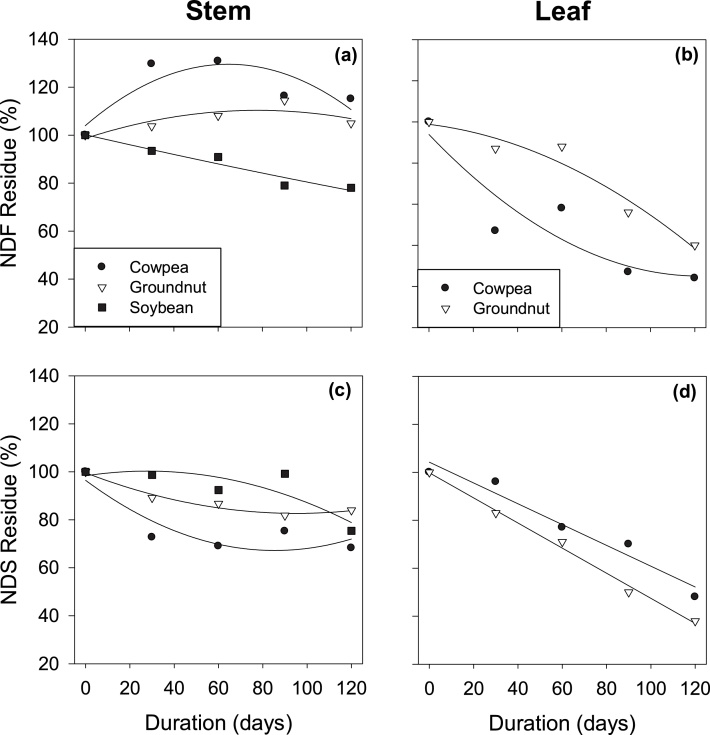
Effect of storage duration on the neutral detergent fibre (NDF) residue and neutral detergent soluble (NDS) residue of stem (a, c) and leaf (b, d) fractions of grain legume fodders as percentage of dry matter residue (DMR) of grain legume fodders at different storage locations and in different types of packaging.

Mean neutral detergent fibre residue (NDFR) of leaf and stem fractions differed among crops and packaging types (Table 3). There was a location effect on NDFR of the leaf fractions where room storage had higher NDFR than storage on tree-fork. There was a duration effect with interactions between duration and crops, duration and packaging type for both leaf and stem fractions. (Table 3; Figs. 5a and b). NDFR of the cowpea stem fraction increased by 15 %, that of groundnut by 5 % while that of soybean reduced by 22 % after 120 days of storage (Fig. 5). At the end of storage, however, NDFR of the leaf fraction reduced in cowpea by 76 % and in groundnut by 60 % (Fig. 5).

**Table 3:** Mean neutral detergent fibre residues (NDFR) and neutral detergent soluble residues (NDSR) of leaf and stem fractions of grain legume fodders stored at different storage locations and in different types of packaging for 120 days.

Treatments	NDFR (%)		NDSR (%)	
	Stem	Leaf	Stem	Leaf
**Crop (C)**				
Cowpea	118^c^	51^b^	77^b^	78^a^
Groundnut	106^b^	74^a^	88^a^	69^b^
Soybean	88^a^		93c	
*P*-value	<0.001	<0.001	<0.001	<0.001
LSD	4.9	6.8	3.5	4.5

**Location (L)**				
Rooftop	103	61^ab^	86	73^ab^
Room	104	69^a^	87	78^a^
Tree-fork	106	58^b^	86	69^b^
*P*-value	ns	0.034	ns	0.014
LSD	4.9	8.4	3.5	5.5

**Packaging (P)**				
Sack	107^a^	73^a^	91^a^	80^a^
Tied	102^b^	52^b^	82^b^	69^b^
*P*-value	<0.029	<0.001	<0.001	<0.001
LSD	3.9	6.8	2.8	4.5

*P*-values for duration (D)				
D	<0.001	<0.001	<0.001	<0.001
D x C	<0.001	0.001	<0.001	0.022
D x L	ns	ns	ns	ns
D x P	<0.001	0.014	<0.001	0.009

Means with different letters in a column of each treatment factor are significantly different (p < 0.05). Leaf only applies to cowpea and groundnut fodders because soybean fodder contained no leaves.

Mean neutral detergent soluble residue (NDSR) of leaf and stem fractions generally reduced among crop and packaging types. Room storage had a higher NDSR in leaf than storage on tree-fork. Sack storage had a lower NDSR than tied fodder in both stem fraction (9 and 18 %, respectively) and leaf fraction (20 % and 31 %, respectively) (Table 3). There was a duration effect with interactions between duration and crop, and between duration and packaging types (Table 3). In contrast to NDFR, stem NDSR of the cowpea stem fraction reduced by 32 %, that of groundnut by 16 % and soybean by 25 % after 120 days of storage (Fig. 5). At the end of storage, however, NDSR of the leaf fraction was reduced in cowpea by 52 % and in groundnut by 62 % (Fig. 5).

### 3.2 Aflatoxin in groundnut fodder

All groundnut samples analysed for aflatoxin contamination showed no detectable levels (parts per billion) of any of the toxins in the samples.

## 4 Discussion

In the present study, differences in pre-storage nutritional quality were observed among crops (Table 1). Soybean fodder had a lower CP content and OMD, and a higher content of cell wall components (NDF, ADF ADL and cellulose) than cowpea and groundnut stems and leaves. These results are typical of these crops at harvest (Anele et al., 2010; Dada et al., 1999; Larbi et al., 1999). These nutritional differences are largely due to variation in the maturity stage of the crops at the time of harvest. In the present study, groundnut was the first crop to be harvested followed by cowpea. Both crops were green and included leaves at the time of harvest. Soybean, on the other hand, was harvested at an advanced stage of maturity when almost all leaves had fallen. These results were in line with the observations by Rinne et al. (1997) and Coleman and Moore (2003), who reported increasing cell wall and decreasing CP contents and OMD with increasing maturity. The groundnut varieties in our study were dual-purpose varieties, i.e. developed for grain and forage production, which may explain the higher LSR of 0.49 of the varieties in our study than the LSR of 0.34 of the varieties in the study of Larbi et al. (1999). The higher cell wall contents in stems than in leaves is in line with studies by Feyissa et al. (2014); Larbi et al. (1999); and Schiere et al. (2004).

The results of the present study showed that storage conditions affected the quantity and nutritional quality of GLFs. On average, DM quantity reduced by 24 % across all storage conditions, with a range from 14 % in the best condition (sacks and in rooms) to 35 % in the worst condition (bundles tied with rope and stored on roofs or on tree-forks (Table 2, Fig. 2). Our present study shows that part of the DM loss can be prevented by storing GLFs in sacks instead of tying bundles with rope, and to a minor extent, in rooms instead of in the open air (Coblentz et al., 2013; Guerrero et al., 2010).This reduction in DM can be attributed to two processes. First, respiration and microbial digestion can convert NDS into volatile components, and fungal activity may even degrade part of the NDF (Nayan et al., 2018). Second, due to drying, brittle plant parts may pulverize and be blown away in the wind.

In stem fractions of GLFs, respiration seemed the most important process in the present study, because NDSR reduced at a higher rate than NDFR indicating that losses should be attributed to respiration and microbial digestion. NDS consists of cell contents which are metabolized during respiration or digested by micro-organisms. Respiration and microbial digestion of NDS may have been facilitated by the high ambient temperature (Fig. 1) at the experimental site (Coblentz et al., 2013; Guerrero et al., 2010; Shayo and Udén, 1999) as well as by the relatively early physiological stage of harvest for cowpea and groundnut. NDFR in stems even increased. It is unknown whether this is observation is caused by measurement errors or by recovery of fungal matter in the NDF. Fungal cell walls consist of chitin which is insoluble in the neutral detergent used for NDF analysis (Nayan et al., 2018; Zhao et al., 2015).

The rate of reduction of NDFR in leaves was comparable to that of NDSR in leaves. This parallel reduction in NDFR and NDSR could imply that pulverization may have caused this loss of leaves. The storage period occurred during the dry season of the year (Fig. 1) and was characterised by no precipitation, low relative humidity and high temperatures. These weather conditions may have promoted the faster rates of drying and pulverization of the brittle leaf fractions of GLFs during storage (Shinners et al., 2010). However, it cannot be excluded that respiration and microbial activity caused part of the loss of leaves too, or facilitated the pulverization. The increase in the cell wall components (NDF and ADF) of cowpea and groundnut in our study corroborates the results of Feyissa et al. (2014) and Guerrero et al. (2010). These authors worked on hays from a natural pasture and alfalfa (*Medicago sativa*), respectively, and reported that prolonged storage of these forages was associated with an increase in content of cell wall components. The high content of cell wall components in a feed is negatively correlated with OMD (Feyissa et al., 2014; Larbi et al., 1999) and dry matter intake in ruminants (Oosting, 1993).

Our study also shows that nutritional quality (CP content and OMD) of cowpea and groundnut reduced most during the first 30 days of storage, while the content of cell wall components increased in the same period (Fig. 3, Fig. 4, Table S1 and S2). These observations can also be explained by the relatively high losses of NDS, which is the fraction with the highest CP content and the highest digestibility (Oosting, 1993). The initial difference between crops had reduced after storage: nevertheless, soybean fodder remained the worst, whereas groundnut had the highest CP content and cowpea the best OMD. The differences in CP content and OMD between cowpea and groundnut agreed with Konlan et al. (2018) and Samireddypalle et al. (2017), who also found high CP content and low OMD in groundnut while the reverse was found in cowpea during a survey of feed markets in Nigeria and Ghana. The nutritive value of soybean fodder was relatively stable during storage when compared with cowpea and groundnut fodder, but remained the lowest. Due to the poor nutritional quality of soybean fodder, including the low intake, it is rarely used for livestock feeding (FAO, 2014; Samireddypalle et al., 2017). The low nutritive quality of soybean fodder (Table 2; Maheri-Sis et al., 2011; Wang et al., 2014) suggests the need to breed dual-purpose soybean varieties for food and feed in the future.

Additionally, GLFs stored in rooms and sacks are of better nutritional quality than those stored in treefook and rooftop and tied with rope (Table 2, Table 3). These results are in line with the findings of Feyissa et al. (2014) and Guerrero et al. (2010), who found that storage conditions are the main factors responsible for DM and nutritional loss or retention during storage. They further stated that loss in DM and nutritional quality is more and faster when hays are stored outdoor and unprotected from adverse weather conditions. According to (Guerrero et al., 2010), unprotected hays stored under high temperatures experience further drying compared to hay tarpaulin covered hays stored under shade.

The absence of aflatoxin in our groundnut fodder samples indicated that it could be used as livestock feed without negative health implications when stored under dry and hot conditions. The prevalence of aflatoxin in animal feed (especially in groundnut and its products) is of great concern for livestock producers, so further research is suggested to ensure aflatoxin does not develop in fodders stored under more moist conditions.

## 5 Conclusion

This paper shows that storage conditions affected the quantity of the dry matter of stored GLFs and the nutritional quality of GLFs. We found that dry matter loss during storage for 120 days was on average 24 % across all storage conditions, 35 % for the worst condition (tied in bundles and stored on roofs or tree-forks) and 14 % for the best condition (sacks and in rooms). During storage, the CP content and OMD decreased, and the content of cell wall components increased. The reduction of nutritional quality was lowest when GLFs were stored in sacks. Storage in sacks and to a lesser extent, storage in rooms (indoor) may reduce the loss of DM and nutritive quality during storage compared to tying in bundles with rope and outdoor storage. Soybean fodder had lower nutritional quality than cowpea and groundnut fodder. The absence of aflatoxin in the groundnut fodder samples indicated that there is no risk of aflatoxin development when stored under dry conditions as in our study.

## Authorship statement

D.B. Akakpo designed the research, collected, analysed data and wrote the paper. I.J.M de Boer and K.E. Giller contributed to the designing of the research and offered technical input in shaping of the paper. S. Adjei-Nsiah supervised the field experimentation and contributed to writing this paper. A.J. Duncan contributed to the conceptualization of the research problem, offered technical advice on the general framework of the paper. S.J. Oosting designed and contributed in the discussion of the results in the paper. He reviewed and offered technical advice on the general framework of the paper.

## Declaration of Competing Interest

The authors declare no conflict of interest.

## References

[bib0005] Akakpo D.B., Oosting S.J., Adjei-Nsiah S., Duncan A., de Boer I.J., Giller K.E. (2020). Do inoculation and Phosphorus Fertilization of Grain Legumes Improve Yield and Fodder Quality? Evidence from the Guinea Savanna of Ghana.

[bib0010] Amole T.A., Ayantunde A., Duncan A.J. (2014). Assessment of existing and potential feed resources to improve livestock productivity in resources to improve livestock. Int. Livest. Res. Inst..

[bib0015] Anele U.Y., Arigbede O.M., Südekum K.H., Ike K.A., Oni A.O., Olanite J.A., Amole G.A., Dele P.A., Jolaosho A.O. (2010). Effects of processed cowpea (Vigna unguiculata L. Walp) haulms as a feed supplement on voluntary intake, utilization and blood profile of West African dwarf sheep fed a basal diet of Pennisetum purpureum in the dry season. Anim. Feed Sci. Technol..

[bib0020] AOAC (1990). Official Methods of Analysis.

[bib0025] Ayantunde A.A., Delfosse P., Fernandez-Rivera S., Gerard B., Dan-Gomma A. (2007). Supplementation with groundnut haulms for sheep fattening in the West African Sahel. Trop. Anim. Health Prod..

[bib0030] Ayantunde A.A., Blummel M., Grings E., Duncan A.J. (2014). Price and quality of livestock feeds in suburban markets of West Africa’s Sahel: case study from Bamako. Anim. Health Prod..

[bib0035] Coblentz W.K., Coffey K.P., Young A.N., Bertram M.G. (2013). Storage characteristics, nutritive value, energy content, and in vivo digestibility of moist, large rectangular bales of alfalfa-orchardgrass hay treated with a propionic acid-based preservative. J. Dairy Sci..

[bib0040] Cole R.J., Dorner J.W. (1994). Extraction of aflatoxins from naturally contaminated peanuts with different solvents and solvent/peanut ratios. J. AOAC Int..

[bib0045] Coleman S.W., Moore J.E. (2003). Feed quality and animal performance. F. Crop. Res..

[bib0050] Dada S.A.O., Adeneye J.A., Akinsoyinu A.O., Smith J.W., Dashiell K.E. (1999). Performance of sheep fed soybean stover and cassava crumb based diets. Small Rumin. Res..

[bib0055] de Boever J.L., Cottyn B.G., Vanacker J.M., Boucqué C.V. (1995). The use of NIRS to predict the chemical composition and the energy value of compound feeds for cattle. Anim. Feed Sci. Technol..

[bib0060] FAO (2014). Crop Residues and Agro-industrial By-products in West Africa - Situation and Way Forward for Livestock Production.

[bib0065] Feyissa F., Prasad S., Assefa G., Bediye S., Kitaw G., Kehaliew A., Kebede G. (2014). Dynamics in nutritional characteristics of natural pasture hay as affected by harvesting stage, storage method and storage duration in the cooler tropical highlands. African J. Agric. Res. Full Length Res. Pap. Afr. J. Agric. Res.

[bib0070] Gallo A., Giuberti G., Frisvad J.C., Bertuzzi T., Nielsen K.F. (2015). Review on mycotoxin issues in ruminants: occurrence in forages, effects of mycotoxin ingestion on health status and animal performance and practical strategies to counteract their negative effects. Toxins (Basel)..

[bib0075] Guerrero J.N., Calderón-Cortés J.F., Montaño-Gómez M.F., González-Vizcarra V., López-Soto M.A. (2010). Effect of storage system and tarpaulin color on nutritional quality and digestibility of stored lucerne hay in the irrigated Sonoran Desert. Anim. Feed Sci. Technol..

[bib0080] Konlan S.P., Ayantunde A.A., Addah W., Dei H.K., Karbo N. (2018). Emerging feed markets for ruminant production in urban and peri-urban areas of Northern Ghana. Trop. Anim. Health Prod..

[bib0085] Larbi A., Dung D.D., Olorunju P.E., Smith J.W., Tanko R.J., Muhammad I.R., Adekunle I.O. (1999). Groundnut (Arachis hypogaea) for food and fodder in crop-livestock systems: forage and seed yields, chemical composition and rumen degradation of leaf and stem fractions of 38 cultivars. Anim. Feed Sci. Technol..

[bib0090] Lemus R. (2009). Hay Storage: Dry Matter Losses and Quality Changes.

[bib0095] López S., Davies D.R., Giráldez F.J., Dhanoa M.S., Dijkstra J., France J. (2005). Assessment of nutritive value of cereal and legume straws based on chemical composition and in vitro digestibility. J. Sci. Food Agric..

[bib0100] Maheri-Sis N., Abdollahi-Ziveh B., Salamatdoustnobar R., Ahmadzadeh A., Aghajanzadeh-Golshani A., Mohebbizadeh M. (2011). Determining nutritive value of soybean straw for ruminants using nylon bags technique. Pak. J. Nutr.

[bib0105] Mertens D.R. (2009). Impact of NDF content and digestibility on dairy cow performance. WCDS Adv. Dairy Technol..

[bib0110] Nayan N., Sonnenberg A.S.M., Hendriks W.H., Cone J.W. (2018). Screening of white-rot fungi for bioprocessing of wheat straw into ruminant feed. J. Appl. Microbiol..

[bib0115] Oosting S.J. (1993). Wheat Straw as Ruminant Feed: Effect of Supplementation and Ammonia Treatment.

[bib0120] Rinne M., Jaakkola S., Huhtanen P. (1997). Grass maturity effects on cattle fed silage-based diets. 1. Organic matter digestion, rumen fermentation and nitrogen utilization. Anim. Feed Sci. Technol..

[bib0125] Samireddypalle A., Boukar O., Grings E., Fatokun C.A., Kodukula P., Devulapalli R., Okike I., Blümmel M. (2017). Cowpea and groundnut haulms fodder trading and its lessons for multidimensional cowpea improvement for mixed crop livestock systems in West Africa. Front. Plant Sci..

[bib0130] Schiere J.B., Joshi A.L., Seetharam A., Oosting S.J., Goodchild A.V., Deinum B., Van Keulen H. (2004). Grain and straw for whole plant value: Implications for crop management and genetic improvement strategies. Exp. Agric..

[bib0135] Searle S.R., Casella G., McCulloch C.E. (1992). Variance Component.

[bib0140] Shayo C.M., Udén P. (1999). Nutritional uniformity of neutral detergent solubles in some tropical browse leaf and pod diets. Anim. Feed Sci. Technol..

[bib0145] Shinners K.J., Boettcher G.C., Muck R.E., Weimer P.J., Casler M.D. (2010). Harvest and storage of two perennial grasses as biomass feedstocks. Trans. Asabe.

[bib0150] Van Soest P.J., Robertson J. (1985). Analysis of Forages and Fibrous Foods.

[bib0155] VSN (2017). Genstat for Windows.

[bib0160] Wang C., Song E., Wang Z., Liu X., Nian H., Zhang J. (2014). Variations in the nutritive value of soybean straw and their use with agronomic traits for breeding assays. J. Anim. Plant Sci..

[bib0165] Woomer P., Huising J., Giller K., Kantengwa S., Boahen S., Wolf De J., Franke L., Abaidoo R., Dianda M., Sanginga J.M., Ronner E., Den Van G. (2013). N2Africa : Final Report of the First Phase N2Africa Putting Nitrogen Fixation to Work for Smallholder Farmers in Africa.

[bib0170] Zhao X., Gong J., Zhou S., OuYang K., Song X., Fu C., Xu L., Qu M. (2015). Effect of fungal treatments of rape straw on chemical composition and in vitro rumen fermentation characteristics. BioResources.

