# Supplementary material for: Evaluating the effects of storage conditions on dry matter loss and nutritional quality of grain legume fodders in West Africa
Source: Anim Feed Sci Technol. 2020 Apr;262:114419. doi: 10.1016/j.anifeedsci.2020.114419 (PMC7104892; doi:10.1016/j.anifeedsci.2020.114419)
Supplement: Supplementary file 1 [file mmc1.docx]

**Table S1**:

Effects of storage duration on dry matter residues (DMR), leaf-to-stem ratio (LSR), crude protein (CP) and organic matter digestibility of grain legume fodders packaged differently with their linear and quadratic levels of significance.

| Crop | Duration | DMR  (%) | LSR^[[1]](#footnote-1)^ | Sack storage (g kg^-1^) | |  | DMR (%) | LSR | Tied storage(g kg^-1^) | |
| --- | --- | --- | --- | --- | --- | --- | --- | --- | --- | --- |
|  |  |  |  | CP | OMD |  |  |  | CP | OMD |
| Cowpea | 0 | 100 | 0.42 | 163 | 742 |  | 100 | 0.42 | 163 | 742 |
|  | 30 | 95.6 | 0.39 | 128 | 703 |  | 92.4 | 0.36 | 117 | 686 |
|  | 60 | 92.3 | 0.34 | 120 | 702 |  | 86.2 | 0.32 | 108 | 651 |
|  | 90 | 89.5 | 0.29 | 128 | 717 |  | 73.7 | 0.21 | 114 | 683 |
|  | 120 | 85.7 | 0.24 | 116 | 682 |  | 65.3 | 0.19 | 108 | 684 |
|  | Mean | 92.61 | 0.34 | 131 | 709.2 |  | 83.5 | 0.30 | 122.0 | 689.2 |
|  | RMSE | 0.48 | 0.05 | 12.97 | 16.14 |  | 1.65 | 0.026 | 17.2 | 31.1 |
|  | *P values* |  |  |  |  |  |  |  |  |  |
|  | Linear | <0.001 | 0.007 | 0.11 | 0.14 |  | 0.001 | 0.003 | 0.129 | 0.313 |
|  | Quadratic | 0.004 | 0.005 | 0.16 | 0.41 |  | 0.070 | 0.036 | 0.111 | 0.131 |
|  |  |  |  |  |  |  |  |  |  |  |
| Groundnut | 0 | 100 | 0.49 | 172 | 660 |  | 100 | 0.49 | 172 | 660 |
|  | 30 | 98.3 | 0.48 | 146 | 678 |  | 91.2 | 0.39 | 145 | 667 |
|  | 60 | 93.1 | 0.45 | 152 | 665 |  | 87.4 | 0.37 | 149 | 658 |
|  | 90 | 89.9 | 0.3 | 147 | 659 |  | 81.1 | 0.21 | 128 | 646 |
|  | 120 | 86.1 | 0.24 | 138 | 667 |  | 65.8 | 0.18 | 128 | 657 |
|  | Mean | 93.5 | 0.39 | 151 | 665.8 |  | 85.1 | 0.33 | 144.4 | 657.6 |
|  | RMSE | 0.94 | 0.36 | 8.2 | 8.7 |  | 3.13 | 0.035 | 8.5 | 7.2 |
|  | *P values* |  |  |  |  |  |  |  |  |  |
|  | Linear | 0.001 | 0.017 | 0.082 | 0.868 |  | 0.006 | 0.005 | 0.030 | 0.322 |
|  | Quadratic | 0.013 | 0.049 | 0.254 | 0.937 |  | 0.030 | 0.005 | 0.120 | 0.674 |
|  |  |  |  |  |  |  |  |  |  |  |
| Soybean | 0 | 100 |  | 97 | 548 |  | 100 |  | 97 | 548 |
|  | 30 | 98.9 |  | 98 | 579 |  | 91.7 |  | 104 | 588 |
|  | 60 | 95.0 |  | 96 | 580 |  | 87.8 |  | 103 | 575 |
|  | 90 | 92.3 |  | 100 | 602 |  | 79.9 |  | 99 | 581 |
|  | 120 | 85.4 |  | 95 | 547 |  | 68.9 |  | 98 | 559 |
|  | Mean | 94.3 |  | 97.2 | 571.2 |  | 85.6 |  | 100.2 | 570.2 |
|  | RMSE | 0.85 |  | 2.19 | 16.4 |  | 1.85 |  | 2.46 | 11.51 |
|  | *P values* |  |  |  |  |  |  |  |  |  |
|  | Linear | 0.007 |  | 0.792 | 0.821 |  | 0.002 |  | 0.807 | 0.816 |
|  | Quadratic | 0.011 |  | 0.799 | 0.243 |  | 0.012 |  | 0.312 | 0.246 |

RMSE: root mean square error,

**Table S2**

Effect of storage duration on the fibre content: neutral detergent fibre (NDF), acid detergent fibre (ADF) and acid detergent lignin (ADL) of grain legume packaged differently with their linear and quadratic levels of significance.

| Crop | Duration | Sack storage (g kg^-1^) | | |  | Tied storage(g kg^-1^) | | |
| --- | --- | --- | --- | --- | --- | --- | --- | --- |
|  |  | NDF | ADF | ADL |  | NDF | ADF | ADL |
| Cowpea | 0 | 446 | 319 | 81 |  | 446 | 319 | 81 |
|  | 30 | 514 | 416 | 88 |  | 562 | 460 | 94 |
|  | 60 | 525 | 427 | 83 |  | 587 | 486 | 95 |
|  | 90 | 502 | 404 | 77 |  | 543 | 457 | 85 |
|  | 120 | 533 | 447 | 86 |  | 551 | 460 | 87 |
|  | **Mean** | **504** | **402.6** | **83** |  | **537.8** | **436.4** | **88.4** |
|  | RMSE | 26.6 | 35.5 | 5.0 |  | 51.6 | 57.7 | 6.90 |
|  | *P values* |  |  |  |  |  |  |  |
|  | Linear | 0.150 | 0.118 | 0.953 |  | 0.326 | 0.224 | 0.899 |
|  | Quadratic | 0.272 | 0.241 | 0.990 |  | 0.188 | 0.122 | 0.451 |
|  |  |  |  |  |  |  |  |  |
| Groundnut | 0 | 422 | 383 | 94 |  | 422 | 383 | 94 |
|  | 30 | 451 | 418 | 97 |  | 476 | 437 | 104 |
|  | 60 | 470 | 433 | 103 |  | 489 | 451 | 109 |
|  | 90 | 485 | 444 | 103 |  | 537 | 490 | 111 |
|  | 120 | 466 | 433 | 95 |  | 506 | 464 | 101 |
|  | Mean | **458.8** | **422.2** | **98.4** |  | **486** | **445** | **103.8** |
|  | RMSE | 6.2 | 2.9 | 4.8 |  | 25.6 | 14.2 | 1.8 |
|  | *P values* |  |  |  |  |  |  |  |
|  | Linear | 0.098 | 0.007 | 0.634 |  | 0.066 | 0.065 | 0.401 |
|  | Quadratic | 0.033 | 0.007 | 0.170 |  | 0.920 | 0.064 | 0.036 |
|  |  |  |  |  |  |  |  |  |
| Soybean | 0 | 652 | 550 | 105 |  | 652 | 550 | 105 |
|  | 30 | 628 | 535 | 110 |  | 650 | 556 | 128 |
|  | 60 | 646 | 548 | 111 |  | 668 | 564 | 124 |
|  | 90 | 588 | 499 | 103 |  | 610 | 521 | 107 |
|  | 120 | 670 | 568 | 114 |  | 648 | 542 | 109 |
|  | Mean | **636.8** | **540** | **108.6** |  | **645.6** | **546.6** | **114.6** |
|  | RMSE | 35.7 | 30.4 | 4.8 |  | 23.1 | 16.5 | 10.4 |
|  | *P values* |  |  |  |  |  |  |  |
|  | Linear | 0.974 | 1.00 | 0.521 |  | 0.558 | 0.401 | 0.755 |
|  | Quadratic | 0.659 | 0.698 | 0.843 |  | 0.874 | 0.730 | 0.481 |

RMSE: root mean square error,

1. LSR only applies to cowpea and groundnut fodders because soybean fodder contained no leaves. [↑](#footnote-ref-1)
